# Human Leukocyte Antigen (HLA) Class I Restricted Epitope Discovery in Yellow Fewer and Dengue Viruses: Importance of HLA Binding Strength

**DOI:** 10.1371/journal.pone.0026494

**Published:** 2011-10-19

**Authors:** Ole Lund, Eduardo J. M. Nascimento, Milton Maciel, Morten Nielsen, Mette Voldby Larsen, Claus Lundegaard, Mikkel Harndahl, Kasper Lamberth, Søren Buus, Jérôme Salmon, Thomas J. August, Ernesto T. A. Marques

**Affiliations:** 1 Department of Systems Biology, Center for Biological Sequence Analysis, Technical University of Denmark, Lyngby, Denmark; 2 Division of Experimental Immunology, Institute of Medical Microbiology and Immunology, The Panum Institute, University of Copenhagen, Copenhagen, Denmark; 3 Department of Infectious Diseases and Microbiology, Center for Vaccine Research, University of Pittsburgh, Pittsburgh, Pennsylvania, United States of America; 4 Department of Pharmacology and Molecular Sciences, School of Medicine, Johns Hopkins University, Baltimore, Maryland, United States of America; 5 Centro de Pesquisas Aggeu Magalhaes, Oswaldo Cruz Foundation (FIOCRUZ), Recife, Brazil; Hallym University College of Medicine, Republic of Korea

## Abstract

Epitopes from all available full-length sequences of yellow fever virus (YFV) and dengue fever virus (DENV) restricted by Human Leukocyte Antigen class I (HLA-I) alleles covering 12 HLA-I supertypes were predicted using the *NetCTL* algorithm. A subset of 179 predicted YFV and 158 predicted DENV epitopes were selected using the *EpiSelect* algorithm to allow for optimal coverage of viral strains. The selected predicted epitopes were synthesized and approximately 75% were found to bind the predicted restricting HLA molecule with an affinity, K_D_, stronger than 500 nM. The immunogenicity of 25 HLA-A*02:01, 28 HLA-A*24:02 and 28 HLA-B*07:02 binding peptides was tested in three HLA-transgenic mice models and led to the identification of 17 HLA-A*02:01, 4 HLA-A*2402 and 4 HLA-B*07:02 immunogenic peptides. The immunogenic peptides bound HLA significantly stronger than the non-immunogenic peptides. All except one of the immunogenic peptides had K_D_ below 100 nM and the peptides with K_D_ below 5 nM were more likely to be immunogenic. In addition, all the immunogenic peptides that were identified as having a high functional avidity had K_D_ below 20 nM. A*02:01 transgenic mice were also inoculated twice with the 17DD YFV vaccine strain. Three of the YFV A*02:01 restricted peptides activated T-cells from the infected mice *in vitro*. All three peptides that elicited responses had an HLA binding affinity of 2 nM or less. The results indicate the importance of the strength of HLA binding in shaping the immune response.

## Introduction

The *Flaviviridae* family includes several important human pathogens. These arboviruses include Dengue (DENV), Yellow fever (YFV), West Nile Virus (WNV) and Japanese Encephalites (JEV). All *Flavivirus* are enveloped, single-stranded RNA (+) viruses coding for a polyprotein precursor of approximately 3,400 amino acids, which is cleaved into three structural (capsid, C; precursor membrane and membrane, prM/M; envelope, E) and seven nonstructural proteins (NS1, 2a, 2b, 3, 4a, 4b and 5) [1], [2].

*Flavivirus* infection, in general, elicits long-lasting immunity. Accumulated evidence indicates that neutralizing antibody responses are associated with protection, and increasing evidence suggests that T-cell CD4^+^ and CD8^+^ responses also play critical roles reducing morbidity and mortality. The T-cells can mediate protection against *Flavivirus* by CD4^+^ helper stimulation of B-cells and by direct killing of infected cells by CD8^+^ cytotoxic leukocytes (CTL). CD4^+^ and CD8^+^ cells have been shown to be essential for protection against WNV, and CD8^+^ T-cells have been shown to provide protective immunity against DENV [3], [4], [5], [6], [7], [8] and YFV infections [9], [10].

*Flavivirus*, as other RNA viruses, has a relatively high mutation rate and uses this mechanism to evade the host immune responses. In particular, DENV has four different serotypes and immunological memory responses, either B- or T-cell, have been shown to be involved in DENV pathogeneses.

Only few vaccines are currently available against *Flavivirus*, among these are the attenuated virus vaccines for YFV and the inactive viral protein formulation for JEV. No DENV vaccine is available for clinical use [7]. The virus-based YFV vaccines are associated with several adverse side effects and frequent fatal reactions [11], [12], while the JEV vaccine is relatively weak and prone to induce allergic reactions [13], [14], [15], [16]. Taken together, this indicates the need for development of novel and better *Flavivirus* vaccines.

Recent advances on epitope discovery and antigen delivery technologies have enabled the development of broadly effective epitope based vaccine formulations. Epitope vaccines are potentially safer since they focus the immune response towards defined epitopes associated with protection, thereby minimizing cross-reactions with the potentially pathogenic epitopes [17], [18], [19], [20], [21], [22]. The objective of this study was to identify HLA-I restricted epitopes covering 12 HLA-I supertypes (A1, A2, A3, A24, A26, B7, B8, B27, B 39, B44, B58 and B62) within conserved regions across all four DENV serotypes and YFV strains. The bioinformatics approaches for selection of the epitopes that provide optimal strain coverage were described previously [23] and the HLA-I binding affinity of the selected peptides were determined using conventional biochemical binding assays [24], [25]. In addition, the immunogenicity of the A2, A24 and B7 HLA-I supertype epitopes were determined in HLA-A*02:01, HLA-A*24:01 and HLA-B*07:02 transgenic mice models [1], [2].

## Materials and Methods

### Bioinformatics

The HLA-I epitope predictions were performed on the basis of a dataset consisting of all strains of YFV and DENV that were fully sequenced in May 2006 and available in the GenBank or RefSeq databases. Epitopes restricted to any of the A1, A2, A3, A24, A26, B7, B8, B27, B 39, B44, B58 and B62 supertype representatives were predicted using an inhouse version 1.0 of the *NetCTL* method including prediction for the A26 and B39 supertypes [26]. In the *NetCTL* method, each possible 9mer peptide in a protein is assigned a score based on a combination of proteasomal cleavage, TAP transport efficiency, and HLA-I binding affinity, with the highest weight assigned to HLA-I affinity.

For YFV, we selected the 16 top-scoring 9mer peptides per supertype for each of the 12 fully sequenced YFV genomes: NC_002031 AY640589 AY603338 AY572535 U54798 AF094612 U21055 U21056 X03700 U17067 U17066 X15062. Next, the *EpiSelect* algorithm was used for selecting a number of predicted epitopes, which together constitute a broad coverage of all strains. The *EpiSelect* algorithm has been described in detail previously [23]. Briefly, it aims at selecting a number of epitopes in a way so that the number of selected epitopes in the viral strain with the fewest selected epitopes is as high as possible. Application of the *EpiSelect* algorithm identified 179 unique candidate epitopes, which are present on average in at least 11 out of the 12 genomes.

For DENV, the 15 top-scoring 9mer peptides per supertype were selected for each of the 36 fully sequenced DENV genomes DENV-1: NC_001477 AY762084 AB189121 AY277664 AF311958 AF226685 AY145121 AB074761 U88537, DENV-2: NC_001474 AY702038 AY702035 AY858036 AY744147 AB122020 AB189124 AF169688 AF169685 AF169682 AF169679 AF276619 AF119661 AF022439 AF022436 M29095, DENV-3: AY923865 AY858039 AY648961 AB189127 AY662691 AY099337 AF317645 AY947539, DENV-4: AY762085 AF326573 AF375822. Application of the *EpiSelect* algorithm identified 158 unique candidate epitopes, which are present on average in at least 35 of the 36 genomes.

### Peptides

The 9mer peptides were synthesized by standard 9-fluorenylmethyloxycarbonyl (FMOC) chemistry, and purified by reversed-phase high-performance liquid chromatography (at least 80%, usually >95% purity) and validated by mass spectrometry (Shafer-N, Copenhagen, Denmark). Peptides were distributed at 500 µg/vial and stored lyophilized at −20°C until use. Peptides were dissolved at 2 mg/mL just before use with 10% (v/v) DMSO (Sigma).

### Biochemical peptide-HLA-I binding assay

The biochemical assay for peptide–HLA-I binding was performed as previously described [24], [25]. Briefly, denatured and purified recombinant HLA heavy chains were diluted into a renaturation buffer containing HLA heavy chain, β2-microglobulin and graded concentrations of the test peptide, and incubated at 18°C for 48 h allowing equilibrium to be reached. We have previously demonstrated that denatured HLA molecules can *de novo* fold efficiently, however, only in the presence of appropriate peptides [27]. The concentration of peptide–HLA complexes generated was measured in a quantitative enzyme-linked immunosorbent assay and plotted against the concentration of peptide offered [24]. Because the effective concentration of HLA (3–5 nM) used in these assays is below the equilibrium dissociation constant (KD) of most high-affinity peptide–HLA interactions, the peptide concentration leading to half-saturation of the HLA is a reasonable approximation of the affinity of the interaction. An initial screening procedure was employed whereby a single high concentration (20,000 nM) of peptide was tested. If no complex formation was found, the peptide was assigned as a non-binder to the HLA molecule in question, conversely, if complex formation was found in the initial screening, a full titration of the peptide was performed to determine the affinity of binding to HLA.

### Animals and immunization protocols

Transgenic mice expressing either HLA-A*02:01 [20], HLA-A *24:02 (Lemonier et al., unpublished) or HLA-B*07:02 [21] were used in the immunization protocols. They were bred and maintained in the Johns Hopkins University School of Medicine Animal Facility. Specific pathogen-free colonies were maintained in a helicobacter-negative mice facility. HLA expression of the experimental transgenic mice was evaluated by flow cytometry using anti HLA-A2 PE (BD Biosciences Pharmigen), anti HLA-A24 PE (MBL) or anti HLA-B7 FITC (Abcam). Mice were immunized subcutaneously (tail base) twice, two to three weeks apart, with 100 µL of pool of peptides (1 µg per peptide) dissolved in PBS and emulsified in TiterMax (CytRx Corporation). Control animals were immunized with DMSO dissolved in PBS and emulsified in TiterMax. Different immunization pools were used according to the transgenic mice and the organism by which the epitopes were predicted from (Table S1). For each experiment, nine mice where immunized with the peptide pools, while three naïve mice were used as controls. Immunization with YF vaccine was also performed subcutaneously with three doses of 10^4^ plaque forming units (PFU) of the attenuated virus, at two weeks interval.

The presence of the HLA transgene in each mouse was confirmed by PCR during breading stages. However, the expression of the HLA gene in this strain of mice is unstable and therefore we screened all the mice with immune staining and flow cytometric analyses and selected for the immunization experiments only the mice that presented high levels of HLA protein expression.

### Ethics Statement

All experiments were approved by the Johns Hopkins Animal Care and Use Committee under protocol number MOO7M78.

### ELISPOT assays for enumeration of IFN-γ spot-forming cells (SFC)

Two weeks after the last immunization, the mice were sacrificed and their spleens removed. Splenocytes were isolated and red cells were lysed with ACK buffer (Pierce). CD4^+^ cells were depleted using anti mouse CD4 microbeads and LD columns according to the manufacturer manual (Myltenyl). The CD4-depleted splenocytes were dissolved in complete RPMI 1640 medium (supplemented with 10% v/v fetal bovine serum (FBS), 100 U/mL penicillin/streptomycin, 2 mM Lglutamine, 50 µM 2-mercaptoethanol and 1 M HEPES buffer). IFN-γ ELISPOT assays were performed by using an ELISPOT set according to the manufacturer's protocol (BD Biosciences Pharmingen). Initially, the ELISPOT plates were coated with anti-IFN-γ at 5 µg/mL and incubated at 4°C overnight. The plates were blocked with RPMI 1640 containing 10% FBS for 2 h at room temperature. Then, each peptide was added into ELISPOT plate at 10 µg/mL, 1 µg/mL and 0.1 µg/mL in complete RPMI. After that, CD4-depleted splenocytes from either immunized or control mice were plated at 0.5 to 1×10^6^ cells per well and incubated for 16–18 hs at 37°C, 5% CO_2_. Media only and concanavalin A (Sigma) were used as negative (background) and positive control respectively. The plates were washed and incubated with biotinylated anti-IFN-γ for 2 h at room temperature, followed by HRP-conjugated avidin for 1 h at room temperature. Reactions were developed with AEC substrate (BD Biosciences). IFN-γ spot forming cells (SFC) were enumerated with the Immunospot Series 3B Analyzer ELISPOT reader, using the Immunospot software version 3.0 (Cellular Technologies Ltd). The data shows the number of SFC/10^6^ cells. The peptides were considered T-cell epitopes if (i) they elicited IFN-γ SFC minus background greater than 10; and (ii) IFN-γ SFC minus 2 standard deviations (SD) must be greater than the background (negative control) plus 2 SD.

### YF vaccine

The human YF vaccine, composed of attenuated 17DD YF virus, was a generous gift from Dr. Ricardo Galler (Oswaldo Cruz Foundation, Rio de Janeiro, Brazil). The vaccine was reconstituted with chilled PBS, kept in an ice bath, and used for immunization of mice within 4 h of reconstitution.

### Statistics

Wilcoxon rank sum tests and Fishers exact tests were performed using the commands wilcox.test(), and fisher.test() implemented in R stats package (http://sekhon.berkeley.edu/stats/html/00Index.html).

## Results

### Flavivirus HLA-I binding peptides

*Yellow fever epitopes:* 179 YFV peptides covering 12 HLA-I supertypes (A1, A2, A3, A24, A26, B7, B8, B27, B 39, B44, B58 and B62) were selected using *NetCTL* and *EpiSelect*. 171 of these could be synthesized and were tested for binding to their respective HLA-I. 121 (71%) of these were found to bind to their predicted restricting HLA-I element with a K_D_ stronger than 500 nM. For a peptide to be immunogenic, it is generally considered a prerequisite that it binds its HLA-I molecule with a K_D_ below 500 nM, since it has been shown that more than 90% of the peptides recognized by CD8^+^ T-cells bind with a K_D_ below this threshold [28].

#### Dengue epitopes

**Figure 1:**
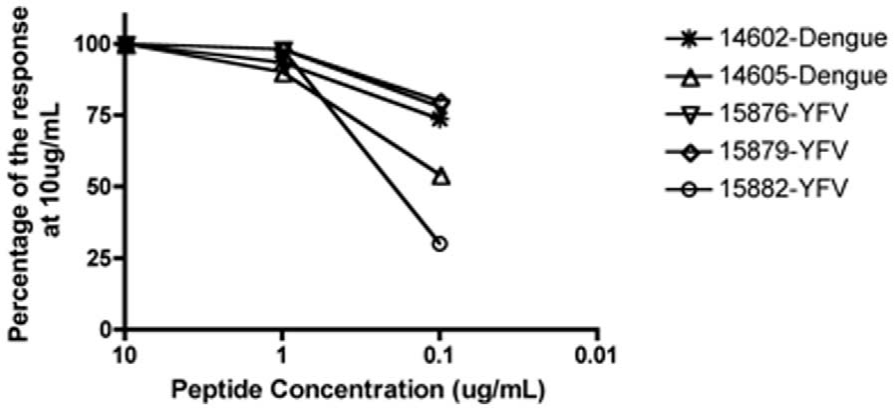
Schematic diagram of the epitope discovery scheme. In addition to the 237 good binders, 5 weak binders and one non-binder was also tested in this study for comparison, but these are not included in the figure.

158 DENV peptides, which together represents all four serotypes and cover the 12 HLA-I supertypes were selected using same procedure as for the YFV epitopes. 147 were synthesized and 116 (79%) were found to bind with a K_D_ below 500 nM. A schematic diagram of the selection scheme is shown in figure 1.

All the peptide sequences along with their binding affinities for the HLA-I alleles were deposited at the Immune Epitope Database (IEDB) (http://www.immuneepitope.org/) and are shown in Table S2.

### Immunogenicity of the A*02:01, A*24:03 and B*07:02 binding peptides

The immunogenicity of peptides binding to representatives of the A2, A24 or B7 HLA-I supertypes with high affinity was tested in transgenic mice models (Table S1). The animals were immunized with these peptides and the T-cell immune responses tested individually by ELISPOT assays as described in materials and methods. The immunogenicity of 82 peptides from both YFV and DENV comprising 25 A*02:01, 28 A*24:03, 28 B*07:02 binders and one non-binding peptide were tested.

Among the A*02:01 binding peptides, 17/25 (68%) were able to elicit a significant immune response under this immunization protocol in at least two individual animals (Table 1). No significant responses were observed in negative control mice or against the HLA non-binding peptide (LVAGGLLTV). The binding affinity was found to be significantly correlated with eliciting responses (p = 0.01, Wilcoxon test). When considering the peptides with a measured affinity below 5 nM to HLA-A*02:01, 12/14 (86%) of the peptides elicited a response which was significantly more than the 5/12 (42%) of the poorer binders (p<0.05, Fishers exact test).

**Table 1:** ELISPOT results on peptide pool-immunized A*02:01 mice.

Peptide (ID)	KD (nM)	Organism	Average number of SFC/million in CD4-depleted splenocytes
LLLTLLATV (14597)	43	DENV	28±21
KMDIGVPLL (14598)	1	DENV	112±19
SMVNGVVRL (14599)	2	DENV	57±28
IMAVGLVSL (14601)	92	DENV	56±13
ILTDGPERV (14602)	4	DENV	170±81
VLNPYMPTV (14604)	1	DENV	166±32
SMVNGVVKL (14605)	25	DENV	125±46
TLYAVATTV (14606)	1	DENV	81±27
VLAPYMPDV (15870)	1	YFV	35±49
IIMDEAHFL (15871)	2	YFV	40±57
YLIIGILTL (15872)	25	YFV	36±35
YMPDVLEKL (15875)	2	YFV	66±68
GLFGGLNWI (15876)	1	YFV	20±2
LLDKQQFEL (15877)	2	YFV	55±4
IMGAVLIWV (15878)	59	YFV	124±59
VLAGWLFHV (15879)	1	YFV	64±71
WMIHTLEAL (15881)	1	YFV	72±54

DENV-Dengue Virus; YFV-Yellow fever virus; NS-Non structural protein; C-Capside; PrM-Pre Membrane; E-Envelop.

The results shown is the average and standard deviation of spot forming cells (SFC)/million splenocytes from two or more mice tested in two or more separate experiments. All peptides shown were considered positive in at least two mice according to the criteria described in materials and methods.

Among the A*24:03 binding peptides, 4/28 (14%) were able to elicit a significant immune response in at least two individual animals (Table 2). We were not able to use exactly the same A24 allele for the binding assays and the animal studies, but the specificity of the two molecules is predicted to be very similar (http://www.cbs.dtu.dk/biotools/MHCMotifViewer/). When immunizing using the peptides with an affinity below 5 nM to HLA-A*24:03, 1/3 (33%) elicited a significant response, whereas a response was observed for only 3/25 (12%) of the peptides binding with an affinity above 5 nM. Here, we hence also find that the likelihood of inducing a CTL response was highest for the strongest binding peptides. The difference between the two groups was not, however, found to be statistically significant (p = 0.27, Fishers exact test).

**Table 2:** ELISPOT results on peptide pool-immunized A*24:02 mice.

Peptide	KD (nM)	Organism	Average number of SFC/million in CD4-depleted splenocytes
WYMWLGARF	19	DENV	119±16
MYADDTAGW	415	DENV	128±6
TYLALMATF	4	DENV	40±15
IFFFLFNIL	19	YFV	14±4

DENV-Dengue Virus; YFV-Yellow fever virus; NS-Non structural protein; C-Capside; PrM-Pre Membrane; E-Envelope.

The results shown is the average and standard deviation of spot forming cells (SFC)/million splenocytes from two or more mice tested in two or more separate experiments. All peptides shown were considered positive in at least two mice according to the criteria described in materials and methods.

Among the B*07:02 peptides, 4/28 (14%) were able to elicit detectable responses in the B*07:02 mice (Table 3). None of the five peptides with binding affinities below 5 nM induced an immune response. Three of the fourteen peptides (21%) with affinities between 5 and 100 nM induced immune responses. The correlation between affinity and immune response was neither statistically significant when using Wilcoxons test, nor Fishers exact test with at affinity threshold of 5 nM. One of the nine peptides with a binding affinity above 100 nM elicited a response, however, it appears to be an outlier, since the peptide's measured affinity for the B*07:02 molecule is 6,769 nM. The peptide was originally predicted to bind to B*07:02 with an affinity of 328 nM. Using the latest version 2.4 of *NetMHCpan* (/www.cbs.dtu.dk/services/NetMHCpan/), the peptide is predicted to bind to B*07:02 with an affinity of 3,529 nM. Given this very low binding affinity to B*07:02 it seems likely that this peptide is restricted by another peptide presenting molecule. Discarding this outlier, we find for B*07:02 that 3 out of the 19 peptides with an affinity stronger than 100 nM elicit a response where this is the case for none of the 9 peptides with an affinity weaker than 100 nM. The fraction of responding peptides as a function of the affinity is summarized in table 4.

**Table 3:** ELISPOT results on peptide pool-immunized B*07:02 mice.

Peptide	KD (nM)	Organism	Average number of SFC/million in CD4-depleted splenocytes
HPGFTILAL	8	DENV	56±40
AVSRGTAKL	6769	YFV	110±42
SPGRKNGSF	27	YFV	205±50
RVKLSALTL	41	YFV	46±20

The results shown is the average and standard deviation of spot forming cells (SFC)/million splenocytes from two or more mice tested in two or more separate experiments. All peptides shown were considered positive in at least two mice according to the criteria described in materials and methods.

**Table 4:** Summary of ELISPOT results on peptide pool-immunized A*02:01, B*07:02 and A*24:02 mice.

Allele	affinity<5 nM	5 nM< = affinity<100 nM	Affinity>100 nM
A*02:01	12/14	5/8	0/4
A*24:03	1/2	2/21	1/5
B*07:02	0/4	3/15	1/9

For each allele and affinity range the number of responders are shown together with the number of tested peptides (after the slash).

It is known that antigen processing plays an important role in shaping the immune dominance. To investigate the effect of processing on the YFV epitopes, the A*02:01 mice were inoculated twice with a dose of 10^4^ PFU of 17DD YFV strain, which is attenuated, but able to replicate in mice. The spleens of the inoculated mice were harvested and the significant T-cell responses evaluated by ELISPOT using the selected A*02:01 peptides (Table 5). Of the three YFV A*02:01 peptides that activated the T-cells from the infected mice *in vitro*, two had previously been shown to be immunogenic and the third, which did not show consistent results with the peptide immunization, now appeared in three separate assays. All three peptides that elicited responses had an HLA-I binding affinity of 2 nM or less. The peptides eliciting responses had stronger HLA binding affinities (average 1.3 nM) than those that did not give a response (average 156 nM). This difference was, however, only borderline significant (p = 0.068; Wilcoxon rank sum test). The peptides that elicited a response when using the 17DD YFV strain to vaccinate also had stronger HLA binding affinities than those that gave response when using A*02:01 peptides from YFV to vaccinate (average 47 nM) but this difference was not significant (p = 0.23; Wilcoxon rank sum test). A similar experiment was also performed in the B*07:02 mice, but no T-cell responses were detected in the B*07:02 mice inoculated with the 17DD virus. A similar experiment was not performed in A*24:02 mice since, unfortunately, these mice were no longer available in our laboratory.

**Table 5:** ELISPOT results on 17DD YFV vaccine-immunized A*02:01 mice.

Peptide	KD (nM)	Organism	Average number of SFC/million in CD4-depleted splenocytes
GLFGGLNWI	1	YFV	402±20
VLAGWLFHV	1	YFV	67±25
GLYGNGILV	2	YFV	18±5

The results shown is the average and standard deviation of spot forming cells (SFC)/million splenocytes of two or more mice tested in two or more separate experiments. All peptides shown were considered positive in at least two mice according to the criteria described in materials and methods.

**Figure 2:**
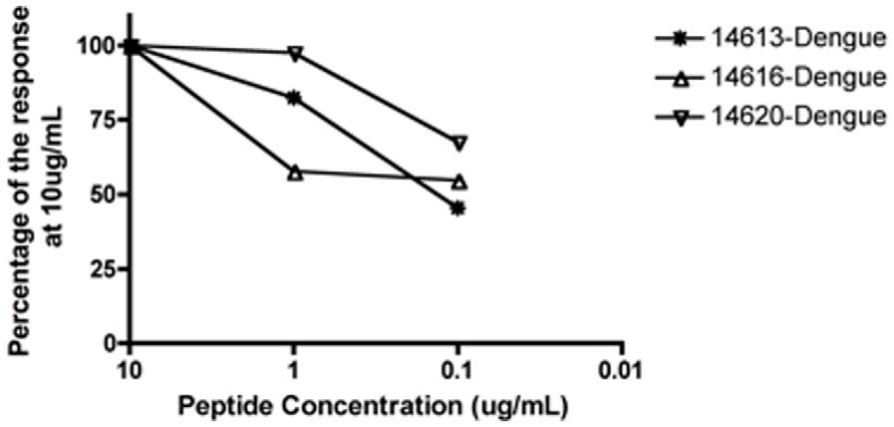
ELISPOT results in 9 mice vaccinated with either A*02:01 dengue peptide pool or YFV 17DD vaccine in A*02:01 mice. Peptides positive at concentration 0.1 µg/mL and greater are shown. Results are depicted as percentage of spot-forming cells/million cells of each peptide at 10 µg/mL.

**Figure 3:**
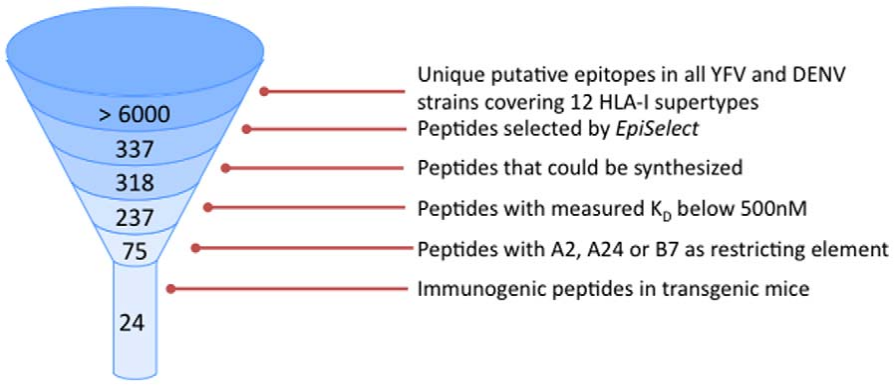
ELISPOT results in 9 mice vaccinated with A*24:03 dengue peptide pool in A*24:02 mice. Peptides positive at concentration 0.1 µg/mL and greater are shown. Results are depicted as percentage of spot-forming cells/million cells of each peptide at 10 µg/mL.

When the peptides giving significant responses were tested *in vitro* at different concentrations, five peptides (two DENV and three YFV) that induced responses in A*02:01 mice showed high functional avidity achieving approximately half the maximal of the T-cell responses at peptide concentrations as low as 0.1 µg/mL (Figure 2). The A*02:01 response was specific, since no peptide from the B*07:02 set (used as negative controls) induced any response in A*02:01 mice (data not shown). Three of the A*24:03 immunogenic peptides elicited high affinity responses (Figure 3). Note that all the peptides that were found to be immunogenic in transgenic B*07:02 mice only activated CTLs at the highest concentration used, 10 µg/ml. Since we have only made graphs of peptides that elicited a response at 0.1 µg/ml, they were not included in any figure.

## Discussion

In this study, we identified 179 yellow fever and 158 dengue peptides as putative HLA-I binders using the *NetCTL* algorithm. Approximately 75% of these peptides bound to at least one of the 12 representatives for the HLA-I supertypes with a K_D_ below 500 nM. The immunogenicity of 25 HLA-A*02:01, 28 HLA-A*24:03 and 28 HLA-B*07:02 binders was tested in three HLA-transgenic mice models and led to the identification of 17 HLA-A*02:01, 4 HLA-A*24:03 and 4 HLA-B*07:02 immunogenic peptides. All except two of the immunogenic peptides had K_D_ below 100 nM, and the peptides with IC_50_ below 5 nM were more likely to be immunogenic. In addition, all the immunogenic peptides that were identified as having a high functional avidity (induce more then 50% of maximum response with 0.1 µg/ml of peptide) had K_D_ below 20 nM.

Considering all three HLA-I types and all 82 tested peptides together, 22 peptides had K_D_ below 5 nM and 13 (59%) of those were immunogenic. Considering the 64 peptides that had affinities to their respective HLA below 100 nM, 23 (36%) induced immune responses, whereas only two of seventeen (12%) of the peptides with K_D_ above 100 nM induced immune responses. For each of the three HLA-I molecules included in this study, we observe an inverse correlation between the likelihood of a peptide binder of inducing a CD8 response and the binding affinity. However, this correlation between the peptide-HLA binding affinity and immunogenicity in the respective transgenic mice model appears to differ according to the HLA type. Among the three HLA-I types evaluated, A*02:01 showed the highest correlation between HLA binding affinity and immunogenicity, since 86% of the peptides with K_D_ below 5 nM induced responses. The reason we did not see a significant correlation for A*24:03 and B*07:02 may be that we found relatively fewer responding peptides for these alleles as compared to A*02:01.

These differences in correlation between HLA binding affinity and immunogenicity may be due to differences in the levels of expression of the HLA molecule in the transgenic mice model or other artifacts of these artificial animal models. However, it could also be due to intrinsic differences on the forces of interactions of specific HLA alleles with the CD8 molecules or with the T-cell receptor that would lead to different requirements of MHC-peptide affinities for optimum T-cell activation. If this is true, it is possible that different HLA alleles preferentially induce responses to peptides with distinct ranges of MHC-peptide binding affinities. This latter speculation is in accordance with earlier observations by Rao et al. [29].

In conclusion, we have described the predicted top binding peptides, all highly conserved in YFV and DENV, for the 12 HLA-I supertypes (see table S2). The potential immunogenicity of these peptides was evaluated in mice models transgenic for three different HLA types and more than half of the peptides with K_D_ below 5 nM for the HLA-1 molecule in question were indeed immunogenic.

## Supporting Information

Table S1 **Peptides analyzed and immunization pools.**
(DOC)

Table S2 **Binding affinities for YFV and DFV peptides.**
(DOC)
