# Correction: Human Leukocyte Antigen (HLA) Class I Restricted Epitope Discovery in Yellow Fewer and Dengue Viruses: Importance of HLA Binding Strength

**DOI:** 10.1371/annotation/254c9f39-1c6b-4f48-a947-b4ed931642e7

**Published:** 2011-11-09

**Authors:** Ole Lund, Eduardo J. M. Nascimento, Milton Maciel, Morten Nielsen, Mette Voldby Larsen, Claus Lundegaard, Mikkel Harndahl, Kasper Lamberth, Søren Buus, Jérôme Salmon, Thomas J. August, Ernesto T. A. Marques

The word "Fever" is misspelled in the title. The correct title is: Human Leukocyte Antigen (HLA) Class I Restricted Epitope Discovery in Yellow Fever and Dengue Viruses: Importance of HLA Binding Strength

The correct citation is: Lund O, Nascimento EJM, Maciel M Jr, Nielsen M, Voldby Larsen M, et al. (2011) Human Leukocyte Antigen (HLA) Class I Restricted Epitope Discovery in Yellow Fever and Dengue Viruses: Importance of HLA Binding Strength. PLoS ONE 6(10): e26494. doi:10.1371/journal.pone.0026494 

