# Supplementary material for: Human Leukocyte Antigen (HLA) Class I Restricted Epitope Discovery in Yellow Fewer and Dengue Viruses: Importance of HLA Binding Strength
Source: PLoS One. 2011 Oct 19;6(10):e26494. doi: 10.1371/journal.pone.0026494 (PMC3198402; doi:10.1371/journal.pone.0026494)
Supplement: Table S1 — Peptides analyzed and immunization pools. (DOC) [file pone.0026494.s001.doc]

Table S1.

| **Immunization Pool** | **Peptide ID** | **HLA** | **KD (nM)** | **Supertype** | **Sequence** | **Start Position** | **Protein/virus** |
| --- | --- | --- | --- | --- | --- | --- | --- |
| **Pool Dengue A2** | 14596 | A*02:01 | 20000 | A2 | LVAGGLLTV | 1376 | NS2B/DENV |
|  | 14597 | A*02:01 | 43 | A2 | LLLTLLATV | 2148 | NS4A/DENV |
|  | 14598 | A*02:01 | 1 | A2 | KMDIGVPLL | 2328 | NS4B/DENV |
|  | 14599 | A*02:01 | 2 | A2 | SMVNGVVRL | 2809 | NS5/DENV |
|  | 14600 | A*02:01 | 48 | A2 | PLNEGIMAV | 1346 | NS2B/DENV |
|  | 14601 | A*02:01 | 92 | A2 | IMAVGLVSL | 1351 | NS2B/DENV |
|  | 14602 | A*02:01 | 4 | A2 | ILTDGPERV | 1906 | NS3/DENV |
|  | 14604 | A*02:01 | 1 | A2 | VLNPYMPTV | 2668 | NS5/DENV |
|  | 14605 | A*02:01 | 25 | A2 | SMVNGVVKL | 2805 | NS5/DENV |
|  | 14606 | A*02:01 | 1 | A2 | TLYAVATTV | 2283 | NS4B/DENV |
|  | 14607 | A*02:01 | 1 | A2 | YMPSVVETL | 2677 | NS5/DENV |
| **Pool Dengue A24** | 14610 | A*24:03 | 13 | A24 | MALVAFLRF | 47 | C/DENV |
|  | 14611 | A*24:03 | 6 | A24 | GWGNGCGLF | 379 | E/DENV |
|  | 14612 | A*24:03 | 119 | A24 | WYGMEIRPL | 1104 | NS1/DENV |
|  | 14613 | A*24:03 | 19 | A24 | WYMWLGARF | 2965 | NS5/DENV |
|  | 14614 | A*24:03 | 61 | A24 | WLGARFLEF | 2968 | NS5/DENV |
|  | 14615 | A*24:03 | 34 | A24 | GFLNEDHWF | 2980 | NS5/DENV |
|  | 14616 | A*24:03 | 415 | A24 | MYADDTAGW | 3020 | NS5/DENV |
|  | 14617 | A*24:03 | 22 | A24 | DYMPSMKRF | 3371 | NS5/DENV |
|  | 14618 | A*24:03 | 4 | A24 | TYGWNLVKL | 2604 | NS5/DENV |
|  | 14619 | A*24:03 | 81 | A24 | IWEVEDYGF | 926 | NS1/DENV |
|  | 14620 | A*24:03 | 4 | A24 | TYLALMATF | 1199 | NS2A/DENV |
|  | 14621 | A*24:03 | 10 | A24 | QYSDRRWCF | 2031 | NS3/DENV |
|  | 14622 | A*24:03 | 17 | A24 | GFMNEDHWF | 2981 | NS5/DENV |
|  | 14623 | A*24:03 | 76 | A24 | TYGWNIVKL | 2607 | NS5/DENV |
| **Pool Dengue B7** | 14708 | B*07:02 | 12 | B7 | SPSKLASAI | 812 | NS1/DENV |
|  | 14709 | B*07:02 | 10 | B7 | LPAIVREAI | 1678 | NS3/DENV |
|  | 14710 | B*07:02 | 11 | B7 | NPITLTAAL | 2345 | NS4B/DENV |
|  | 14711 | B*07:02 | 755 | B7 | EPKEGTKKL | 2855 | NS5/DENV |
|  | 14712 | B*07:02 | 444 | B7 | RERLSRMAI | 3142 | NS5/DENV |
|  | 14713 | B*07:02 | 2 | B7 | ILRNPGFAL | 239 | PrM/DENV |
|  | 14714 | B*07:02 | 18 | B7 | IPLCRTSCL | 1302 | NS2A/DENV |
|  | 14715 | B*07:02 | 7 | B7 | RVIDPRRCL | 1894 | NS3/DENV |
|  | 14716 | B*07:02 | 14 | B7 | QPKPGTRMV | 2851 | NS5/DENV |
|  | 14717 | B*07:02 | 30 | B7 | MPVMKRYSA | 3370 | NS5/DENV |
|  | 14718 | B*07:02 | 6 | B7 | GPRMKLVMAF | 41 | C/DENV |
|  | 14719 | B*07:02 | 821 | B7 | CPTQGEATL | 353 | E/DENV |
|  | 14720 | B*07:02 | 3 | B7 | GPSLRTTTV | 1069 | NS1/DENV |
|  | 14721 | B*07:02 | 3 | B7 | YPRMSIPATL | 1454 | NS2B/DENV |
|  | 14722 | B*07:02 | 8 | B7 | HPGFTILAL | 243 | PrM/DENV |
| **Pool YFV A2** | 15870 | A*02:01 | 1 | A2 | VLAPYMPDV | 2688 | NS5/YFV |
|  | 15871 | A*02:01 | 3 | A2 | IIMDEAHFL | 1769 | NS3/YFV |
|  | 15872 | A*02:01 | 25 | A2 | YLIIGILTL | 2241 | NS4A/YFV |
|  | 15873 | A*02:01 | 49 | A2 | TFWMGSHEV | 976 | NS1/YFV |
|  | 15875 | A*02:01 | 2 | A2 | YMPDVLEKL | 2692 | NS5/YFV |
|  | 15876 | A*02:01 | 1 | A2 | GLFGGLNWI | 728 | E/YFV |
|  | 15877 | A*02:01 | 2 | A2 | LLDKQQFEL | 2523 | NS5/YFV |
|  | 15878 | A*02:01 | 59 | A2 | IMGAVLIWV | 740 | E/YFV |
|  | 15879 | A*02:01 | 1 | A2 | VLAGWLFHV | 1470 | NS2B/YFV |
|  | 15880 | A*02:01 | 325 | A2 | VMAPDKPSL | 317 | E/YFV |
|  | 15881 | A*02:01 | 1 | A2 | WMIHTLEAL | 988 | NS1/YFV |
|  | 15882 | A*02:01 | 2 | A2 | GLYGNGILV | 1634 | NS3/YFV |
|  | 15883 | A*02:01 | 114 | A2 | AMLHWSLIL | 2371 | NS4B/YFV |
|  | 15884 | A*02:01 | 1251 | A2 | FVRNPFFAV | 245 | M/YFV |
|  | 16029 | A*02:01 | 39 | A2 | KMLDPRQGL | 69 | C/YFV |
| **Pool YFV A24** | 15900 | A*24:03 | 19 | A24 | IFFFLFNIL | 47 | C/YFV |
|  | 15903 | A*24:03 | 17 | A24 | RWFVRNPFF | 243 | M/YFV |
|  | 15904 | A*24:03 | 13 | A24 | MAGCGYLMF | 2193 | NS4A/YFV |
|  | 15905 | A*24:03 | 32 | A24 | MWHVTRGAF | 1534 | NS3/YFV |
|  | 15906 | A*24:03 | 35 | A24 | WYMWLGARY | 2981 | NS5/YFV |
|  | 15907 | A*24:03 | 9 | A24 | MYMALIAAF | 1201 | NS2A/YFV |
|  | 15908 | A*24:03 | 303 | A24 | VMGDAAWDF | 696 | E/YFV |
|  | 15909 | A*24:03 | 5 | A24 | YYAFVGVMY | 2487 | NS4B/YFV |
|  | 15910 | A*24:03 | 373 | A24 | IHTVFGSAF | 718 | E/YFV |
|  | 15911 | A*24:03 | 35 | A24 | WLGARYLEF | 2984 | NS5/YFV |
|  | 15912 | A*24:03 | 14 | A24 | AFHGLDVKF | 1722 | NS3/YFV |
|  | 15913 | A*24:03 | 6 | A24 | GWGNGCGLF | 384 | E/YFV |
|  | 16031 | A*24:03 | 6 | A24 | VYQRGTHPF | 873 | NS1/YFV |
|  | 16032 | A*24:03 | 253 | A24 | VYMDAVFEY | 944 | NS1/YFV |
| **Pool YFV B7** | 15926 | B*07:02 | 3 | B7 | MVRRGVRSL | 14 | C/YFV |
|  | 15928 | B*07:02 | 6769 | B7 | AVSRGTAKL | 2559 | NS5/YFV |
|  | 15929 | B*07:02 | 42 | B7 | RPAPGGKAY | 3088 | NS5/YFV |
|  | 15930 | B*07:02 | 272 | B7 | TPFGQQRVF | 2853 | NS5/YFV |
|  | 15931 | B*07:02 | 5 | B7 | MPRSIGGPV | 1020 | NS1/YFV |
|  | 15932 | B*07:02 | 27 | B7 | SPGRKNGSF | 902 | NS1/YFV |
|  | 15933 | B*07:02 | 6 | B7 | RAYRNALSM | 2144 | NS4A/YFV |
|  | 15934 | B*07:02 | 95 | B7 | RVSSDQSAL | 2087 | NS3/YFV |
|  | 15935 | B*07:02 | 41 | B7 | RVKLSALTL | 568 | E/YFV |
|  | 15936 | B*07:02 | 761 | B7 | QVVMTSLAL | 1449 | NS2B/YFV |
|  | 15937 | B*07:02 | 117 | B7 | GPAEARKVC | 336 | E/YFV |
|  | 16035 | B*07:02 | 240 | B7 | RVLDCRTAF | 1909 | NS3/YFV |
|  | 16036 | B*07:02 | 290 | B7 | SVAMCRTPF | 2438 | NS4B/YFV |

DENV-Dengue Virus; YFV-Yellow fever virus; NS-Non structural protein; C-Capside; PrM-Pre Membrane; E-Envelope
