# Supplementary material for: Human Leukocyte Antigen (HLA) Class I Restricted Epitope Discovery in Yellow Fewer and Dengue Viruses: Importance of HLA Binding Strength
Source: PLoS One. 2011 Oct 19;6(10):e26494. doi: 10.1371/journal.pone.0026494 (PMC3198402; doi:10.1371/journal.pone.0026494)
Supplement: Table S2 — Binding affinities for YFV and DFV peptides. (DOC) [file pone.0026494.s002.doc]

Table S2.

| Batch Number | Name | Peptide Sequence | HLA Name | KdValue (nM) |
| --- | --- | --- | --- | --- |
| 16028 | YFV [A1/A26/B62] | EISGSSARY | A*0101 | 95 |
| 16027 | YFV [A1/A26] 16027 | SSHNHIPGY | A*0101 | 847 |
| 15859 | YFV [A1] 15859 | RVERIKSEY | A*0101 | 20000 |
| 15867 | YFV [A1] 15867 | AVTALTIAY | A*0101 | 990 |
| 15869 | YFV [A1] 15869 | GVQGFIFFF | A*0101 | 20000 |
| 15858 | YFV A1 15858 | NRDGDSYYY | A*0101 | 1924 |
| 15860 | YFV A1 15860 | HSEEGSRAY | A*0101 | 14 |
| 15861 | YFV A1 15861 | GVEGIGLQY | A*0101 | 48 |
| 15862 | YFV A1 15862 | DSDDWLNKY | A*0101 | 1 |
| 15863 | YFV A1 15863 | MTGVMRGNY | A*0101 | 54 |
| 15864 | YFV A1 15864 | YANMWSLMY | A*0101 | 1 |
| 15865 | YFV A1 15865 | MNNGGDAMY | A*0101 | 796 |
| 15866 | YFV A1 15866 | NTDIKTLKF | A*0101 | 4 |
| 16025 | YFV A1/A26 16025 | GTGDSRLTY | A*0101 | 75 |
| 16026 | YFV A1/A26 16026 | EVDQTKIQY | A*0101 | 166 |
| 15872 | YFV [A2] 15872 | YLIIGILTL | A*0201 | 25 |
| 15876 | YFV [A2] 15876 | GLFGGLNWI | A*0201 | 1 |
| 15870 | YFV A2 15870 | VLAPYMPDV | A*0201 | 1 |
| 15871 | YFV A2 15871 | IIMDEAHFL | A*0201 | 3 |
| 15873 | YFV A2 15873 | TFWMGSHEV | A*0201 | 49 |
| 15875 | YFV A2 15875 | YMPDVLEKL | A*0201 | 2 |
| 15877 | YFV A2 15877 | LLDKQQFEL | A*0201 | 2 |
| 15878 | YFV A2 15878 | IMGAVLIWV | A*0201 | 59 |
| 15879 | YFV A2 15879 | VLAGWLFHV | A*0201 | 1 |
| 15880 | YFV A2 15880 | VMAPDKPSL | A*0201 | 325 |
| 15881 | YFV A2 15881 | WMIHTLEAL | A*0201 | 1 |
| 15882 | YFV A2 15882 | GLYGNGILV | A*0201 | 2 |
| 15883 | YFV A2 15883 | AMLHWSLIL | A*0201 | 114 |
| 15884 | YFV A2 15884 | FVRNPFFAV | A*0201 | 1251 |
| 16029 | YFV A2/B62 16029 | KMLDPRQGL | A*0201 | 39 |
| 15893 | YFV [A3] 15893 | VVMQVKVPK | A*0301 | 363 |
| 15885 | YFV A3 15885 | LTLKGTSYK | A*0301 | 104 |
| 15886 | YFV A3 15886 | TAHLKRLWK | A*0301 | 1322 |
| 15887 | YFV A3 15887 | ALNTITNLK | A*0301 | 245 |
| 15888 | YFV A3 15888 | VLDCRTAFK | A*0301 | 75 |
| 15889 | YFV A3 15889 | KTALTGAMR | A*0301 | 147 |
| 15890 | YFV A3 15890 | TINAVASRK | A*0301 | 129 |
| 15891 | YFV A3 15891 | MVIFFMSPK | A*0301 | 610 |
| 15892 | YFV A3 15892 | KTHESHLVR | A*0301 | 678 |
| 15894 | YFV A3 15894 | VLWDIPTPK | A*0301 | 14 |
| 15895 | YFV A3 15895 | GTHPFSRIR | A*0301 | 859 |
| 15896 | YFV A3 15896 | RMGERQLQK | A*0301 | 340 |
| 15897 | YFV A3 15897 | RVDGLELKK | A*0301 | 203 |
| 15898 | YFV A3 15898 | VMYNLWKMK | A*0301 | 4 |
| 15899 | YFV A3 15899 | KVVNRWLFR | A*0301 | 218 |
| 16030 | YFV A3/B62 16030 | RSHAAIGAY | A*0301 | 313 |
| 16032 | YFV [A24/B62] 16032 | VYMDAVFEY | A*2403 | 253 |
| 15900 | YFV [A24] 15900 | IFFFLFNIL | A*2403 | 19 |
| 15903 | YFV [A24] 15903 | RWFVRNPFF | A*2403 | 17 |
| 15910 | YFV [A24] 15910 | IHTVFGSAF | A*2403 | 373 |
| 15901 | YFV A24 15901 | WFYDNDNPY | A*2403 | 5100 |
| 15902 | YFV A24 15902 | LVGAAIHPF | A*2403 | 575 |
| 15904 | YFV A24 15904 | MAGCGYLMF | A*2403 | 13 |
| 15905 | YFV A24 15905 | MWHVTRGAF | A*2403 | 32 |
| 15906 | YFV A24 15906 | WYMWLGARY | A*2403 | 35 |
| 15907 | YFV A24 15907 | MYMALIAAF | A*2403 | 9 |
| 15908 | YFV A24 15908 | VMGDAAWDF | A*2403 | 303 |
| 15909 | YFV A24 15909 | YYAFVGVMY | A*2403 | 5 |
| 15911 | YFV A24 15911 | WLGARYLEF | A*2403 | 35 |
| 15912 | YFV A24 15912 | AFHGLDVKF | A*2403 | 14 |
| 16031 | YFV A24/B8 16031 | VYQRGTHPF | A*2403 | 6 |
| 16028 | YFV [A1/A26/B62] | EISGSSARY | A*2601 | 3 |
| 16027 | YFV [A1/A26] 16027 | SSHNHIPGY | A*2601 | 136 |
| 16033 | YFV [A26/B62] 16033 | SVKEDLVAY | A*2601 | 21 |
| 15920 | YFV [A26] 15920 | FLILGMLLM | A*2601 | 20000 |
| 16025 | YFV A1/A26 16025 | GTGDSRLTY | A*2601 | 20000 |
| 16026 | YFV A1/A26 16026 | EVDQTKIQY | A*2601 | 3685 |
| 15914 | YFV A26 15914 | FCVKVLAPY | A*2601 | 427 |
| 15915 | YFV A26 15915 | EAMDTISVF | A*2601 | 7 |
| 15916 | YFV A26 15916 | ETACLSKAY | A*2601 | 8 |
| 15917 | YFV A26 15917 | YVIRDLAAM | A*2601 | 1 |
| 15918 | YFV A26 15918 | EAPEMPALY | A*2601 | 594 |
| 15919 | YFV A26 15919 | TAVDFGNSY | A*2601 | 8 |
| 15921 | YFV A26 15921 | LVALTLTSY | A*2601 | 1160 |
| 15922 | YFV A26 15922 | DVKFHTQAF | A*2601 | 61 |
| 15923 | YFV A26 15923 | YTIDCDGSI | A*2601 | 251 |
| 16034 | YFV A26/B62 16034 | LVLAVGPAY | A*2601 | 5119 |
| 15893 | YFV [A3] 15893 | VVMQVKVPK | A*3101 | 15 |
| 15885 | YFV A3 15885 | LTLKGTSYK | A*3101 | 518 |
| 15891 | YFV A3 15891 | MVIFFMSPK | A*3101 | 1396 |
| 15896 | YFV A3 15896 | RMGERQLQK | A*3101 | 187 |
| 15953 | YFV B27 15953 | RQEKWMTGR | A*3101 | 7 |
| 15936 | YFV [B7] 15936 | QVVMTSLAL | B*0702 | 761 |
| 15925 | YFV B7 15925 | AATIRVLAL | B*0702 | 204 |
| 15926 | YFV B7 15926 | MVRRGVRSL | B*0702 | 3 |
| 15928 | YFV B7 15928 | AVSRGTAKL | B*0702 | 6769 |
| 15929 | YFV B7 15929 | RPAPGGKAY | B*0702 | 42 |
| 15930 | YFV B7 15930 | TPFGQQRVF | B*0702 | 272 |
| 15931 | YFV B7 15931 | MPRSIGGPV | B*0702 | 5 |
| 15932 | YFV B7 15932 | SPGRKNGSF | B*0702 | 27 |
| 15933 | YFV B7 15933 | RAYRNALSM | B*0702 | 6 |
| 15934 | YFV B7 15934 | RVSSDQSAL | B*0702 | 95 |
| 15935 | YFV B7 15935 | RVKLSALTL | B*0702 | 41 |
| 15937 | YFV B7 15937 | GPAEARKVC | B*0702 | 117 |
| 16035 | YFV B7/B62 16035 | RVLDCRTAF | B*0702 | 240 |
| 16036 | YFV B7/B62 16036 | SVAMCRTPF | B*0702 | 290 |
| 15948 | YFV [B8] 15948 | IMKVVNRWL | B*0801 | 815 |
| 16031 | YFV A24/B8 16031 | VYQRGTHPF | B*0801 | 2371 |
| 15938 | YFV B8 15938 | IGKLFTQTM | B*0801 | 730 |
| 15939 | YFV B8 15939 | QLQKIERWF | B*0801 | 20000 |
| 15940 | YFV B8 15940 | CARRRLRTL | B*0801 | 77 |
| 15941 | YFV B8 15941 | DYKECEWPL | B*0801 | 20000 |
| 15942 | YFV B8 15942 | HLKRLWKML | B*0801 | 16 |
| 15943 | YFV B8 15943 | ALYEKKLAL | B*0801 | 57 |
| 15944 | YFV B8 15944 | SCRVKLSAL | B*0801 | 558 |
| 15945 | YFV B8 15945 | YFHKRDMRL | B*0801 | 60 |
| 15946 | YFV B8 15946 | EVRLATMLF | B*0801 | 1749 |
| 15947 | YFV B8 15947 | EMKEAFHGL | B*0801 | 232 |
| 15949 | YFV B8 15949 | VNRWLFRHL | B*0801 | 20000 |
| 15950 | YFV B8 15950 | AIHPFALLL | B*0801 | 20000 |
| 15951 | YFV B8 15951 | GSRAYRNAL | B*0801 | 694 |
| 15952 | YFV B8 15952 | TLKGTSYKM | B*0801 | 1000 |
| 16018 | YFV [B62] 16018 | RTWHYCGSY | B*1501 | 68 |
| 16023 | YFV [B62] 16023 | VVQDPKNVY | B*1501 | 174 |
| 16017 | YFV B62 16017 | KMCTDKMSF | B*1501 | 184 |
| 16019 | YFV B62 16019 | GMMGGLWKY | B*1501 | 32 |
| 16020 | YFV B62 16020 | SQEAEFTGY | B*1501 | 94 |
| 16021 | YFV B62 16021 | AQAVMEMTY | B*1501 | 56 |
| 16022 | YFV B62 16022 | SIQDNQVAY | B*1501 | 217 |
| 16024 | YFV B62 16024 | VLIEVNPPF | B*1501 | 69 |
| 15968 | YFV [B27] 15968 | RRMRRPTGK | B*2705 | 47 |
| 15953 | YFV B27 15953 | RQEKWMTGR | B*2705 | 2492 |
| 15954 | YFV B27 15954 | KRVVASLMR | B*2705 | 107 |
| 15955 | YFV B27 15955 | WKYLNAVSL | B*2705 | 1419 |
| 15956 | YFV B27 15956 | ARYDVALSE | B*2705 | 20000 |
| 15957 | YFV B27 15957 | RRLRTLVLA | B*2705 | 54 |
| 15959 | YFV B27 15959 | RRVFHGVAK | B*2705 | 14 |
| 15960 | YFV B27 15960 | SRIRDGLQY | B*2705 | 538 |
| 15961 | YFV B27 15961 | SRLTYQWHK | B*2705 | 16 |
| 15962 | YFV B27 15962 | RKVCYNAVL | B*2705 | 548 |
| 15963 | YFV B27 15963 | RRFGGTVIR | B*2705 | 10 |
| 15964 | YFV B27 15964 | SRLLMRRMR | B*2705 | 295 |
| 15965 | YFV B27 15965 | RRRLRTLVL | B*2705 | 111 |
| 15966 | YFV B27 15966 | RQWAQDLTL | B*2705 | 26 |
| 15967 | YFV B27 15967 | YRNALSMMP | B*2705 | 20000 |
| 15867 | YFV [A1] 15867 | AVTALTIAY | B*3501 | 3 |
| 16015 | YFV [B58] 16015 | LAVMGDAAW | B*3501 | 1 |
| 15860 | YFV A1 15860 | HSEEGSRAY | B*3501 | 3 |
| 15864 | YFV A1 15864 | YANMWSLMY | B*3501 | 1 |
| 15865 | YFV A1 15865 | MNNGGDAMY | B*3501 | 46 |
| 15901 | YFV A24 15901 | WFYDNDNPY | B*3501 | 1 |
| 15902 | YFV A24 15902 | LVGAAIHPF | B*3501 | 29 |
| 15904 | YFV A24 15904 | MAGCGYLMF | B*3501 | 2 |
| 15907 | YFV A24 15907 | MYMALIAAF | B*3501 | 207 |
| 15914 | YFV A26 15914 | FCVKVLAPY | B*3501 | 10 |
| 15915 | YFV A26 15915 | EAMDTISVF | B*3501 | 1 |
| 15916 | YFV A26 15916 | ETACLSKAY | B*3501 | 288 |
| 15917 | YFV A26 15917 | YVIRDLAAM | B*3501 | 1 |
| 15919 | YFV A26 15919 | TAVDFGNSY | B*3501 | 1 |
| 16034 | YFV A26/B62 16034 | LVLAVGPAY | B*3501 | 1 |
| 16024 | YFV B62 16024 | VLIEVNPPF | B*3501 | 39 |
| 15972 | YFV [B39] 15972 | YYPEDPVKL | B*3901 | 20000 |
| 15973 | YFV [B39] 15973 | FHERGYVKL | B*3901 | 35 |
| 15974 | YFV [B39] 15974 | RADEINAIL | B*3901 | 156 |
| 15978 | YFV [B39] 15978 | TKDTNDNNL | B*3901 | 136 |
| 15995 | YFV [B44] 15995 | GEAMDTISV | B*3901 | 20000 |
| 15970 | YFV B39 15970 | IQYVIRAQL | B*3901 | 682 |
| 15971 | YFV B39 15971 | ERWFVRNPF | B*3901 | 20000 |
| 15975 | YFV B39 15975 | VRGGMVAPL | B*3901 | 5370 |
| 15976 | YFV B39 15976 | FHGVAKNPV | B*3901 | 60 |
| 15977 | YFV B39 15977 | SHDVLTVQF | B*3901 | 272 |
| 15979 | YFV B39 15979 | CRTAFKPVL | B*3901 | 2059 |
| 15980 | YFV B39 15980 | FHKRDMRLL | B*3901 | 20000 |
| 15981 | YFV B39 15981 | SHAAIGAYL | B*3901 | 23 |
| 15982 | YFV B39 15982 | SSARYDVAL | B*3901 | 377 |
| 15983 | YFV B39 15983 | PHAATIRVL | B*3901 | 960 |
| 15984 | YFV B39 15984 | SRKASNTIL | B*3901 | 525 |
| 15991 | YFV [B44] 15991 | AEMGANLCV | B*4001 | 2 |
| 15995 | YFV [B44] 15995 | GEAMDTISV | B*4001 | 1 |
| 15985 | YFV B44 15985 | HEVNGTWMI | B*4001 | 10 |
| 15986 | YFV B44 15986 | KEVSGVKGF | B*4001 | 109 |
| 15987 | YFV B44 15987 | VQGFIFFFL | B*4001 | 20000 |
| 15988 | YFV B44 15988 | QEGSLKTAL | B*4001 | 2896 |
| 15989 | YFV B44 15989 | IEEFGTGVF | B*4001 | 9 |
| 15990 | YFV B44 15990 | SEQGEFKLL | B*4001 | 140 |
| 15992 | YFV B44 15992 | VEGIGLQYL | B*4001 | 145 |
| 15993 | YFV B44 15993 | YEKKLALYL | B*4001 | 60 |
| 15996 | YFV B44 15996 | AEVRLATML | B*4001 | 24 |
| 15997 | YFV B44 15997 | LEDGIYGIF | B*4001 | 1 |
| 15999 | YFV B44 15999 | REMHHLVEF | B*4001 | 1 |
| 16000 | YFV B44 16000 | QEKWMTGRM | B*4001 | 719 |
| 15931 | YFV B7 15931 | MPRSIGGPV | B*5101 | 858 |
| 16002 | YFV [B58] 16002 | KIMKVVNRW | B*5801 | 37 |
| 16010 | YFV [B58] 16010 | ESWIVDRQW | B*5801 | 123 |
| 16015 | YFV [B58] 16015 | LAVMGDAAW | B*5801 | 3 |
| 16001 | YFV B58 16001 | VSSAVPTSW | B*5801 | 4 |
| 16003 | YFV B58 16003 | KAYANMWSL | B*5801 | 44 |
| 16004 | YFV B58 16004 | KTNDRKWCF | B*5801 | 58 |
| 16005 | YFV B58 16005 | VALSEQGEF | B*5801 | 286 |
| 16006 | YFV B58 16006 | MLSPMLHHW | B*5801 | 8 |
| 16007 | YFV B58 16007 | RQLQKIERW | B*5801 | 67 |
| 16008 | YFV B58 16008 | KTWGKNLVF | B*5801 | 19 |
| 16009 | YFV B58 16009 | KSAHGSPTF | B*5801 | 36 |
| 16011 | YFV B58 16011 | ISYIMLIFF | B*5801 | 1051 |
| 16014 | YFV B58 16014 | LADKRPTAW | B*5801 | 95 |
| 16016 | YFV B58 16016 | ITAHLKRLW | B*5801 | 46 |
| 14581 | Dengue [A1] 14581 | KAELEDGAY | A*0101 | 20000 |
| 14582 | Dengue [A1] 14582 | MANIFRGSY | A*0101 | 20000 |
| 14583 | Dengue [A1] 14583 | KTWAYHGSY | A*0101 | 20000 |
| 14584 | Dengue [A1] 14584 | ETACLGKSY | A*0101 | 1750 |
| 14585 | Dengue [A1] 14585 | YAQMWSLMY | A*0101 | 6 |
| 14586 | Dengue [A1] 14586 | SVEVKLPDY | A*0101 | 690 |
| 14587 | Dengue [A1] 14587 | GTTVVKVKY | A*0101 | 20000 |
| 14588 | Dengue [A1] 14588 | MTTTANWLW | A*0101 | 2336 |
| 14589 | Dengue [A1] 14589 | ETACLGKAY | A*0101 | 20000 |
| 14590 | Dengue [A1] 14590 | KSAAIDGEY | A*0101 | 50 |
| 14591 | Dengue [A1] 14591 | AIDLDPVVY | A*0101 | 25 |
| 14592 | Dengue [A1] 14592 | ESDPEGALW | A*0101 | 57 |
| 14593 | Dengue [A1] 14593 | LMSGKDVFY | A*0101 | 217 |
| 14594 | Dengue [A1] 14594 | YAQMWTLMY | A*0101 | 2 |
| 14596 | Dengue [A2] 14596 | LVAGGLLTV | A*0201 | 20000 |
| 14597 | Dengue [A2] 14597 | LLLTLLATV | A*0201 | 43 |
| 14598 | Dengue [A2] 14598 | KMDIGVPLL | A*0201 | 1 |
| 14599 | Dengue [A2] 14599 | SMVNGVVRL | A*0201 | 2 |
| 14600 | Dengue [A2] 14600 | PLNEGIMAV | A*0201 | 48 |
| 14601 | Dengue [A2] 14601 | IMAVGLVSL | A*0201 | 93 |
| 14602 | Dengue [A2] 14602 | ILTDGPERV | A*0201 | 4 |
| 14604 | Dengue [A2] 14604 | VLNPYMPTV | A*0201 | 1 |
| 14605 | Dengue [A2] 14605 | SMVNGVVKL | A*0201 | 25 |
| 14606 | Dengue [A2] 14606 | TLYAVATTV | A*0201 | 1 |
| 14607 | Dengue [A2] 14607 | YMPSVVETL | A*0201 | 1 |
| 14638 | Dengue [A3] 14638 | TVNPIVTEK | A*0301 | 40 |
| 14639 | Dengue [A3] 14639 | RSADLELER | A*0301 | 842 |
| 14640 | Dengue [A3] 14640 | KTFVDLMRR | A*0301 | 80 |
| 14641 | Dengue [A3] 14641 | AVSMANIFR | A*0301 | 52 |
| 14642 | Dengue [A3] 14642 | SSMVNGVVR | A*0301 | 20000 |
| 14644 | Dengue [A3] 14644 | VVTLIPLCR | A*0301 | 9040 |
| 14645 | Dengue [A3] 14645 | SSADLSLEK | A*0301 | 85 |
| 14646 | Dengue [A3] 14646 | KTFVELMRR | A*0301 | 45 |
| 14647 | Dengue [A3] 14647 | KVASAGISY | A*0301 | 85 |
| 14649 | Dengue [A3] 14649 | ATYGWNLVK | A*0301 | 3 |
| 14650 | Dengue [A3] 14650 | KTFDTEYQK | A*0301 | 198 |
| 14651 | Dengue [A3] 14651 | SYYCAGLKK | A*0301 | 2130 |
| 14652 | Dengue [A3] 14652 | TVMDIISRK | A*0301 | 30 |
| 14610 | Dengue [A24] 14610 | MALVAFLRF | A*2403 | 13 |
| 14611/15913 | Dengue [A24] 14611/YFV A24 15913 | GWGNGCGLF | A*2403 | 6 |
| 14612 | Dengue [A24] 14612 | WYGMEIRPL | A*2403 | 119 |
| 14613 | Dengue [A24] 14613 | WYMWLGARF | A*2403 | 19 |
| 14614 | Dengue [A24] 14614 | WLGARFLEF | A*2403 | 61 |
| 14615 | Dengue [A24] 14615 | GFLNEDHWF | A*2403 | 34 |
| 14616 | Dengue [A24] 14616 | MYADDTAGW | A*2403 | 415 |
| 14617 | Dengue [A24] 14617 | DYMPSMKRF | A*2403 | 22 |
| 14618 | Dengue [A24] 14618 | TYGWNLVKL | A*2403 | 4 |
| 14619 | Dengue [A24] 14619 | IWEVEDYGF | A*2403 | 81 |
| 14620 | Dengue [A24] 14620 | TYLALMATF | A*2403 | 4 |
| 14621 | Dengue [A24] 14621 | QYSDRRWCF | A*2403 | 10 |
| 14622 | Dengue [A24] 14622 | GFMNEDHWF | A*2403 | 17 |
| 14623 | Dengue [A24] 14623 | TYGWNIVKL | A*2403 | 76 |
| 14624 | Dengue [A24] 14624 | WHDWQQVPF | A*2403 | 170 |
| 14625 | Dengue [A26] 14625 | NVHTWTEQY | A*2601 | 39 |
| 14626 | Dengue [A26] 14626 | FTMRLLSPV | A*2601 | 21 |
| 14627 | Dengue [A26] 14627 | ETLETLLLL | A*2601 | 347 |
| 14628 | Dengue [A26] 14628 | FCIKVLNPY | A*2601 | 20000 |
| 14629 | Dengue [A26] 14629 | EYTDYMPSM | A*2601 | 1816 |
| 14630 | Dengue [A26] 14630 | FTTNIWMKF | A*2601 | 117 |
| 14631 | Dengue [A26] 14631 | TTRHRKPTY | A*2601 | 20000 |
| 14632 | Dengue [A26] 14632 | EVHTWTEQY | A*2601 | 2 |
| 14633 | Dengue [A26] 14633 | VTRGAVLMY | A*2601 | 241 |
| 14634 | Dengue [A26] 14634 | SVKKDLISY | A*2601 | 31 |
| 14637 | Dengue [A26] 14637 | DVVPMVTQM | A*2601 | 2 |
| 14583 | Dengue [A1] 14583 | KTWAYHGSY | A*3101 | 20000 |
| 14639 | Dengue [A3] 14639 | RSADLELER | A*3101 | 43 |
| 14667 | Dengue [B27] 14667 | RQLANAIFK | A*3101 | 183 |
| 14704 | Dengue [B62] 14704 | RTWAYHGSY | A*3101 | 37 |
| 14708 | Dengue [B7] 14708 | SPSKLASAI | B*0702 | 12 |
| 14709 | Dengue [B7] 14709 | LPAIVREAI | B*0702 | 11 |
| 14710 | Dengue [B7] 14710 | NPITLTAAL | B*0702 | 11 |
| 14711 | Dengue [B7] 14711 | EPKEGTKKL | B*0702 | 755 |
| 14712 | Dengue [B7] 14712 | RERLSRMAI | B*0702 | 444 |
| 14713 | Dengue [B7] 14713 | ILRNPGFAL | B*0702 | 2 |
| 14714 | Dengue [B7] 14714 | IPLCRTSCL | B*0702 | 18 |
| 14715 | Dengue [B7] 14715 | RVIDPRRCL | B*0702 | 7 |
| 14716 | Dengue [B7] 14716 | QPKPGTRMV | B*0702 | 14 |
| 14717 | Dengue [B7] 14717 | MPVMKRYSA | B*0702 | 30 |
| 14718 | Dengue [B7] 14718 | GPMKLVMAF | B*0702 | 6 |
| 14719 | Dengue [B7] 14719 | CPTQGEATL | B*0702 | 821 |
| 14720 | Dengue [B7] 14720 | GPSLRTTTV | B*0702 | 3 |
| 14721 | Dengue [B7] 14721 | YPMSIPATL | B*0702 | 3 |
| 14722 | Dengue [B7] 14722 | HPGFTILAL | B*0702 | 8 |
| 14723 | Dengue [B8] 14723 | KCRLRMDKL | B*0801 | 6922 |
| 14724 | Dengue [B8] 14724 | QLKGMSYSM | B*0801 | 104 |
| 14725 | Dengue [B8] 14725 | CAHWKEAKM | B*0801 | 7049 |
| 14726 | Dengue [B8] 14726 | RKKLKPRWL | B*0801 | 20000 |
| 14727 | Dengue [B8] 14727 | YFHRRDLRL | B*0801 | 52 |
| 14728 | Dengue [B8] 14728 | IFRKKRLTI | B*0801 | 80 |
| 14729 | Dengue [B8] 14729 | FRKKRLTIM | B*0801 | 14 |
| 14730 | Dengue [B8] 14730 | KKKLRPRWL | B*0801 | 12711 |
| 14731 | Dengue [B8] 14731 | LAKRFSKGL | B*0801 | 4475 |
| 14732 | Dengue [B8] 14732 | KCRLKMDKL | B*0801 | 18296 |
| 14733 | Dengue [B8] 14733 | SPKRLSAAI | B*0801 | 41 |
| 14734 | Dengue [B8] 14734 | RKKLRPRWL | B*0801 | 16907 |
| 14735 | Dengue [B8] 14735 | ELKGMSYAM | B*0801 | 132 |
| 14736 | Dengue [B8] 14736 | VINWKGKEL | B*0801 | 2365 |
| 14737 | Dengue [B8] 14737 | KWKKKLNQL | B*0801 | 20000 |
| 14697 | Dengue [B62] 14697 | KLQLKGMSY | B*1501 | 111 |
| 14698 | Dengue [B62] 14698 | ILGDTAWDF | B*1501 | 274 |
| 14699 | Dengue [B62] 14699 | SQHNYRPGY | B*1501 | 22 |
| 14700 | Dengue [B62] 14700 | TLYAVATTF | B*1501 | 133 |
| 14701 | Dengue [B62] 14701 | ILGETAWDF | B*1501 | 525 |
| 14702 | Dengue [B62] 14702 | AQEDDQYVF | B*1501 | 81 |
| 14703 | Dengue [B62] 14703 | YLAGAGLAF | B*1501 | 11 |
| 14704 | Dengue [B62] 14704 | RTWAYHGSY | B*1501 | 12 |
| 14705 | Dengue [B62] 14705 | KLEGKIVQY | B*1501 | 126 |
| 14706 | Dengue [B62] 14706 | GMGEAAAIF | B*1501 | 29 |
| 14707 | Dengue [B62] 14707 | KQIANELNY | B*1501 | 113 |
| 14653 | Dengue [B27] 14653 | RREKRSVAL | B*2705 | 400 |
| 14654 | Dengue [B27] 14654 | RQWFLDLPL | B*2705 | 32 |
| 14655 | Dengue [B27] 14655 | YRLRGEARK | B*2705 | 86 |
| 14656 | Dengue [B27] 14656 | KRAAAGIMK | B*2705 | 75 |
| 14657 | Dengue [B27] 14657 | KQLGQVMLL | B*2705 | 129 |
| 14658 | Dengue [B27] 14658 | SRAIWYMWL | B*2705 | 2049 |
| 14659 | Dengue [B27] 14659 | FRKGSSIGK | B*2705 | 52 |
| 14660 | Dengue [B27] 14660 | HRLMSAAIK | B*2705 | 18 |
| 14661 | Dengue [B27] 14661 | WRTIMAVLF | B*2705 | 232 |
| 14662 | Dengue [B27] 14662 | RRCLKPVIL | B*2705 | 2928 |
| 14663 | Dengue [B27] 14663 | RRDKRSVAL | B*2705 | 52 |
| 14664 | Dengue [B27] 14664 | SRWSRKMLM | B*2705 | 2 |
| 14665 | Dengue [B27] 14665 | YRILQRGLL | B*2705 | 54 |
| 14666 | Dengue [B27] 14666 | RKHGGMLVR | B*2705 | 348 |
| 14667 | Dengue [B27] 14667 | RQLANAIFK | B*2705 | 28 |
| 14582 | Dengue [A1] 14582 | MANIFRGSY | B*3501 | 829 |
| 14585 | Dengue [A1] 14585 | YAQMWSLMY | B*3501 | 1 |
| 14594 | Dengue [A1] 14594 | YAQMWTLMY | B*3501 | 1 |
| 14688 | Dengue [B58] 14688 | KAVHADMGY | B*3501 | 302 |
| 14703 | Dengue [B62] 14703 | YLAGAGLAF | B*3501 | 4 |
| 14668 | Dengue [B44] 14668 | AELTGYGTV | B*4001 | 21 |
| 14669 | Dengue [B44] 14669 | QEGAMHTAL | B*4001 | 3 |
| 14670 | Dengue [B44] 14670 | GEDGCWYGM | B*4001 | 10 |
| 14671 | Dengue [B44] 14671 | EEEQTLTIL | B*4001 | 2 |
| 14672 | Dengue [B44] 14672 | SEMGANFKA | B*4001 | 496 |
| 14673/15998 | Dengue [B44] 14673/YFV B44 15998 | LEFEALGFL | B*4001 | 1 |
| 14674 | Dengue [B44] 14674 | VEGEGLHKL | B*4001 | 190 |
| 14675 | Dengue [B44] 14675 | AQMWSLMYF | B*4001 | 32 |
| 14676 | Dengue [B44] 14676 | REDQWCGSL | B*4001 | 2 |
| 14677 | Dengue [B44] 14677 | SEHTGREIV | B*4001 | 21 |
| 14678 | Dengue [B44] 14678 | SEMGANFRA | B*4001 | 377 |
| 14679 | Dengue [B44] 14679 | LEFFLMVLL | B*4001 | 177 |
| 14680 | Dengue [B44] 14680 | MEVQLIRQM | B*4001 | 158 |
| 14681 | Dengue [B44] 14681 | REGKIVGLY | B*4001 | 1316 |
| 14682 | Dengue [B44] 14682 | SEHTGKEIV | B*4001 | 16 |
| 14610 | Dengue [A24] 14610 | MALVAFLRF | B*5101 | 20000 |
| 14626 | Dengue [A26] 14626 | FTMRLLSPV | B*5101 | 20000 |
| 14709 | Dengue [B7] 14709 | LPAIVREAI | B*5101 | 621 |
| 14721 | Dengue [B7] 14721 | YPMSIPATL | B*5101 | 295 |
| 14733 | Dengue [B8] 14733 | SPKRLSAAI | B*5101 | 20000 |
| 14683 | Dengue [B58] 14683 | KAWLVHRQW | B*5801 | 18 |
| 14684 | Dengue [B58] 14684 | ITAAAWYLW | B*5801 | 46 |
| 14685 | Dengue [B58] 14685 | ISYGGGWKL | B*5801 | 87 |
| 14686 | Dengue [B58] 14686 | ICSAVPSHW | B*5801 | 394 |
| 14688 | Dengue [B58] 14688 | KAVHADMGY | B*5801 | 30 |
| 14689 | Dengue [B58] 14689 | STMPLVMAW | B*5801 | 3 |
| 14690 | Dengue [B58] 14690 | ISYGGGWRL | B*5801 | 44 |
| 14691 | Dengue [B58] 14691 | KAYAQMWSL | B*5801 | 10 |
| 14692 | Dengue [B58] 14692 | WSIHAHHQW | B*5801 | 7 |
| 14693 | Dengue [B58] 14693 | STTSQKTTW | B*5801 | 31 |
| 14694 | Dengue [B58] 14694 | VSGKLIHEW | B*5801 | 32 |
| 14695 | Dengue [B58] 14695 | ICSAVPVHW | B*5801 | 76 |

Binding affinities for DENV peptides.

| Batch Number | Name | Peptide Sequence | HLA Name | KdValue (nM) |
| --- | --- | --- | --- | --- |
| 14581 | Dengue [A1] 14581 | KAELEDGAY | A*0101 | 20000 |
| 14582 | Dengue [A1] 14582 | MANIFRGSY | A*0101 | 20000 |
| 14583 | Dengue [A1] 14583 | KTWAYHGSY | A*0101 | 20000 |
| 14584 | Dengue [A1] 14584 | ETACLGKSY | A*0101 | 1750 |
| 14585 | Dengue [A1] 14585 | YAQMWSLMY | A*0101 | 6 |
| 14586 | Dengue [A1] 14586 | SVEVKLPDY | A*0101 | 690 |
| 14587 | Dengue [A1] 14587 | GTTVVKVKY | A*0101 | 20000 |
| 14588 | Dengue [A1] 14588 | MTTTANWLW | A*0101 | 2336 |
| 14589 | Dengue [A1] 14589 | ETACLGKAY | A*0101 | 20000 |
| 14590 | Dengue [A1] 14590 | KSAAIDGEY | A*0101 | 50 |
| 14591 | Dengue [A1] 14591 | AIDLDPVVY | A*0101 | 25 |
| 14592 | Dengue [A1] 14592 | ESDPEGALW | A*0101 | 57 |
| 14593 | Dengue [A1] 14593 | LMSGKDVFY | A*0101 | 217 |
| 14594 | Dengue [A1] 14594 | YAQMWTLMY | A*0101 | 2 |
| 14596 | Dengue [A2] 14596 | LVAGGLLTV | A*0201 | 20000 |
| 14597 | Dengue [A2] 14597 | LLLTLLATV | A*0201 | 43 |
| 14598 | Dengue [A2] 14598 | KMDIGVPLL | A*0201 | 1 |
| 14599 | Dengue [A2] 14599 | SMVNGVVRL | A*0201 | 2 |
| 14600 | Dengue [A2] 14600 | PLNEGIMAV | A*0201 | 48 |
| 14601 | Dengue [A2] 14601 | IMAVGLVSL | A*0201 | 93 |
| 14602 | Dengue [A2] 14602 | ILTDGPERV | A*0201 | 4 |
| 14604 | Dengue [A2] 14604 | VLNPYMPTV | A*0201 | 1 |
| 14605 | Dengue [A2] 14605 | SMVNGVVKL | A*0201 | 25 |
| 14606 | Dengue [A2] 14606 | TLYAVATTV | A*0201 | 1 |
| 14607 | Dengue [A2] 14607 | YMPSVVETL | A*0201 | 1 |
| 14610 | Dengue [A24] 14610 | MALVAFLRF | A*2403 | 13 |
| 14611/15913 | Dengue [A24] 14611/YFV A24 15913 | GWGNGCGLF | A*2403 | 6 |
| 14612 | Dengue [A24] 14612 | WYGMEIRPL | A*2403 | 119 |
| 14613 | Dengue [A24] 14613 | WYMWLGARF | A*2403 | 19 |
| 14614 | Dengue [A24] 14614 | WLGARFLEF | A*2403 | 61 |
| 14615 | Dengue [A24] 14615 | GFLNEDHWF | A*2403 | 34 |
| 14616 | Dengue [A24] 14616 | MYADDTAGW | A*2403 | 415 |
| 14617 | Dengue [A24] 14617 | DYMPSMKRF | A*2403 | 22 |
| 14618 | Dengue [A24] 14618 | TYGWNLVKL | A*2403 | 4 |
| 14619 | Dengue [A24] 14619 | IWEVEDYGF | A*2403 | 81 |
| 14620 | Dengue [A24] 14620 | TYLALMATF | A*2403 | 4 |
| 14621 | Dengue [A24] 14621 | QYSDRRWCF | A*2403 | 10 |
| 14622 | Dengue [A24] 14622 | GFMNEDHWF | A*2403 | 17 |
| 14623 | Dengue [A24] 14623 | TYGWNIVKL | A*2403 | 76 |
| 14624 | Dengue [A24] 14624 | WHDWQQVPF | A*2403 | 170 |
| 14625 | Dengue [A26] 14625 | NVHTWTEQY | A*2601 | 39 |
| 14626 | Dengue [A26] 14626 | FTMRLLSPV | A*2601 | 21 |
| 14627 | Dengue [A26] 14627 | ETLETLLLL | A*2601 | 347 |
| 14628 | Dengue [A26] 14628 | FCIKVLNPY | A*2601 | 20000 |
| 14629 | Dengue [A26] 14629 | EYTDYMPSM | A*2601 | 1816 |
| 14630 | Dengue [A26] 14630 | FTTNIWMKF | A*2601 | 117 |
| 14631 | Dengue [A26] 14631 | TTRHRKPTY | A*2601 | 20000 |
| 14632 | Dengue [A26] 14632 | EVHTWTEQY | A*2601 | 2 |
| 14633 | Dengue [A26] 14633 | VTRGAVLMY | A*2601 | 241 |
| 14634 | Dengue [A26] 14634 | SVKKDLISY | A*2601 | 31 |
| 14637 | Dengue [A26] 14637 | DVVPMVTQM | A*2601 | 2 |
| 14638 | Dengue [A3] 14638 | TVNPIVTEK | A*0301 | 40 |
| 14639 | Dengue [A3] 14639 | RSADLELER | A*0301 | 842 |
| 14640 | Dengue [A3] 14640 | KTFVDLMRR | A*0301 | 80 |
| 14641 | Dengue [A3] 14641 | AVSMANIFR | A*0301 | 52 |
| 14642 | Dengue [A3] 14642 | SSMVNGVVR | A*0301 | 20000 |
| 14644 | Dengue [A3] 14644 | VVTLIPLCR | A*0301 | 9040 |
| 14645 | Dengue [A3] 14645 | SSADLSLEK | A*0301 | 85 |
| 14646 | Dengue [A3] 14646 | KTFVELMRR | A*0301 | 45 |
| 14647 | Dengue [A3] 14647 | KVASAGISY | A*0301 | 85 |
| 14649 | Dengue [A3] 14649 | ATYGWNLVK | A*0301 | 3 |
| 14650 | Dengue [A3] 14650 | KTFDTEYQK | A*0301 | 198 |
| 14651 | Dengue [A3] 14651 | SYYCAGLKK | A*0301 | 2130 |
| 14652 | Dengue [A3] 14652 | TVMDIISRK | A*0301 | 30 |
| 14653 | Dengue [B27] 14653 | RREKRSVAL | B*2705 | 400 |
| 14654 | Dengue [B27] 14654 | RQWFLDLPL | B*2705 | 32 |
| 14655 | Dengue [B27] 14655 | YRLRGEARK | B*2705 | 86 |
| 14656 | Dengue [B27] 14656 | KRAAAGIMK | B*2705 | 75 |
| 14657 | Dengue [B27] 14657 | KQLGQVMLL | B*2705 | 129 |
| 14658 | Dengue [B27] 14658 | SRAIWYMWL | B*2705 | 2049 |
| 14659 | Dengue [B27] 14659 | FRKGSSIGK | B*2705 | 52 |
| 14660 | Dengue [B27] 14660 | HRLMSAAIK | B*2705 | 18 |
| 14661 | Dengue [B27] 14661 | WRTIMAVLF | B*2705 | 232 |
| 14662 | Dengue [B27] 14662 | RRCLKPVIL | B*2705 | 2928 |
| 14663 | Dengue [B27] 14663 | RRDKRSVAL | B*2705 | 52 |
| 14664 | Dengue [B27] 14664 | SRWSRKMLM | B*2705 | 2 |
| 14665 | Dengue [B27] 14665 | YRILQRGLL | B*2705 | 54 |
| 14666 | Dengue [B27] 14666 | RKHGGMLVR | B*2705 | 348 |
| 14667 | Dengue [B27] 14667 | RQLANAIFK | B*2705 | 28 |
| 14668 | Dengue [B44] 14668 | AELTGYGTV | B*4001 | 21 |
| 14669 | Dengue [B44] 14669 | QEGAMHTAL | B*4001 | 3 |
| 14670 | Dengue [B44] 14670 | GEDGCWYGM | B*4001 | 10 |
| 14671 | Dengue [B44] 14671 | EEEQTLTIL | B*4001 | 2 |
| 14672 | Dengue [B44] 14672 | SEMGANFKA | B*4001 | 496 |
| 14673/15998 | Dengue [B44] 14673/YFV B44 15998 | LEFEALGFL | B*4001 | 1 |
| 14674 | Dengue [B44] 14674 | VEGEGLHKL | B*4001 | 190 |
| 14675 | Dengue [B44] 14675 | AQMWSLMYF | B*4001 | 32 |
| 14676 | Dengue [B44] 14676 | REDQWCGSL | B*4001 | 2 |
| 14677 | Dengue [B44] 14677 | SEHTGREIV | B*4001 | 21 |
| 14678 | Dengue [B44] 14678 | SEMGANFRA | B*4001 | 377 |
| 14679 | Dengue [B44] 14679 | LEFFLMVLL | B*4001 | 177 |
| 14680 | Dengue [B44] 14680 | MEVQLIRQM | B*4001 | 158 |
| 14681 | Dengue [B44] 14681 | REGKIVGLY | B*4001 | 1316 |
| 14682 | Dengue [B44] 14682 | SEHTGKEIV | B*4001 | 16 |
| 14683 | Dengue [B58] 14683 | KAWLVHRQW | B*5801 | 18 |
| 14684 | Dengue [B58] 14684 | ITAAAWYLW | B*5801 | 46 |
| 14685 | Dengue [B58] 14685 | ISYGGGWKL | B*5801 | 87 |
| 14686 | Dengue [B58] 14686 | ICSAVPSHW | B*5801 | 394 |
| 14688 | Dengue [B58] 14688 | KAVHADMGY | B*5801 | 30 |
| 14689 | Dengue [B58] 14689 | STMPLVMAW | B*5801 | 3 |
| 14690 | Dengue [B58] 14690 | ISYGGGWRL | B*5801 | 44 |
| 14691 | Dengue [B58] 14691 | KAYAQMWSL | B*5801 | 10 |
| 14692 | Dengue [B58] 14692 | WSIHAHHQW | B*5801 | 7 |
| 14693 | Dengue [B58] 14693 | STTSQKTTW | B*5801 | 31 |
| 14694 | Dengue [B58] 14694 | VSGKLIHEW | B*5801 | 32 |
| 14695 | Dengue [B58] 14695 | ICSAVPVHW | B*5801 | 76 |
| 14697 | Dengue [B62] 14697 | KLQLKGMSY | B*1501 | 111 |
| 14698 | Dengue [B62] 14698 | ILGDTAWDF | B*1501 | 274 |
| 14699 | Dengue [B62] 14699 | SQHNYRPGY | B*1501 | 22 |
| 14700 | Dengue [B62] 14700 | TLYAVATTF | B*1501 | 133 |
| 14701 | Dengue [B62] 14701 | ILGETAWDF | B*1501 | 525 |
| 14702 | Dengue [B62] 14702 | AQEDDQYVF | B*1501 | 81 |
| 14703 | Dengue [B62] 14703 | YLAGAGLAF | B*1501 | 11 |
| 14704 | Dengue [B62] 14704 | RTWAYHGSY | B*1501 | 12 |
| 14705 | Dengue [B62] 14705 | KLEGKIVQY | B*1501 | 126 |
| 14706 | Dengue [B62] 14706 | GMGEAAAIF | B*1501 | 29 |
| 14707 | Dengue [B62] 14707 | KQIANELNY | B*1501 | 113 |
| 14708 | Dengue [B7] 14708 | SPSKLASAI | B*0702 | 12 |
| 14709 | Dengue [B7] 14709 | LPAIVREAI | B*0702 | 11 |
| 14710 | Dengue [B7] 14710 | NPITLTAAL | B*0702 | 11 |
| 14711 | Dengue [B7] 14711 | EPKEGTKKL | B*0702 | 755 |
| 14712 | Dengue [B7] 14712 | RERLSRMAI | B*0702 | 444 |
| 14713 | Dengue [B7] 14713 | ILRNPGFAL | B*0702 | 2 |
| 14714 | Dengue [B7] 14714 | IPLCRTSCL | B*0702 | 18 |
| 14715 | Dengue [B7] 14715 | RVIDPRRCL | B*0702 | 7 |
| 14716 | Dengue [B7] 14716 | QPKPGTRMV | B*0702 | 14 |
| 14717 | Dengue [B7] 14717 | MPVMKRYSA | B*0702 | 30 |
| 14718 | Dengue [B7] 14718 | GPMKLVMAF | B*0702 | 6 |
| 14719 | Dengue [B7] 14719 | CPTQGEATL | B*0702 | 821 |
| 14720 | Dengue [B7] 14720 | GPSLRTTTV | B*0702 | 3 |
| 14721 | Dengue [B7] 14721 | YPMSIPATL | B*0702 | 3 |
| 14722 | Dengue [B7] 14722 | HPGFTILAL | B*0702 | 8 |
| 14723 | Dengue [B8] 14723 | KCRLRMDKL | B*0801 | 6922 |
| 14724 | Dengue [B8] 14724 | QLKGMSYSM | B*0801 | 104 |
| 14725 | Dengue [B8] 14725 | CAHWKEAKM | B*0801 | 7049 |
| 14726 | Dengue [B8] 14726 | RKKLKPRWL | B*0801 | 20000 |
| 14727 | Dengue [B8] 14727 | YFHRRDLRL | B*0801 | 52 |
| 14728 | Dengue [B8] 14728 | IFRKKRLTI | B*0801 | 80 |
| 14729 | Dengue [B8] 14729 | FRKKRLTIM | B*0801 | 14 |
| 14730 | Dengue [B8] 14730 | KKKLRPRWL | B*0801 | 12711 |
| 14731 | Dengue [B8] 14731 | LAKRFSKGL | B*0801 | 4475 |
| 14732 | Dengue [B8] 14732 | KCRLKMDKL | B*0801 | 18296 |
| 14733 | Dengue [B8] 14733 | SPKRLSAAI | B*0801 | 41 |
| 14734 | Dengue [B8] 14734 | RKKLRPRWL | B*0801 | 16907 |
| 14735 | Dengue [B8] 14735 | ELKGMSYAM | B*0801 | 132 |
| 14736 | Dengue [B8] 14736 | VINWKGKEL | B*0801 | 2365 |
| 14737 | Dengue [B8] 14737 | KWKKKLNQL | B*0801 | 20000 |
